# Supplementary material for: The EMT transcription factor Snai1 maintains myocardial wall integrity by repressing intermediate filament gene expression
Source: eLife. 2021 Jun 21;10:e66143. doi: 10.7554/eLife.66143 (PMC8216718; doi:10.7554/eLife.66143)
Supplement: Supplementary file 1. [file elife-66143-supp1.docx]

**Table S1. Primer list**

|  | Gene name | Forward | Reverse |  |
| --- | --- | --- | --- | --- |
| PCR | *snai1b* | ATGCCACGCTCATTTCTTGT | GAGCGCCGGACAGCAGCC | To generate the overexpression plasmid |
| PCR | *desmb* | ATGAGCCACTCTTATGCCAC | CATGAGGTCCTGCTGGTG | To generate the overexpression plasmid |
| PCR | 800 bp *desmb* promoter | GAAAGCATAGTCTGCTTTCTCG | GAGCGCCGGACAGCAGCC | To generate the plasmid for the luciferase assay |
| HRM | *snai1b ex1* | ATGCCACGCTCATTTCTTGTCAA | AATTTCACTCTCACCAGTCTGA | Genotyping |
| HRM | *snai1b promoter* | ACCTTCTTGTTGTGAGGCGA | AATTTCACTCTCACCAGTCTGA | Genotyping |
| qPCR | *snai1b* | CAGTGAACTGGAGAGTCAGACTG | CACTGCGGGACGACTGCATA |  |
| qPCR | *snai1a* | CAACTTCAGAGTTCAGCATGC | \|  \| CTGGTGAAGTGTCGTTCTGAC \| \| --- \| --- \| |  |
| qPCR | *desmb* | GGACAACCTGGCAGATGACC | GCCTGCAGCTCACGGATTTC |  |
| qPCR | *rpl13a* | TCTGGAGGACTGTAAGAGGTATGC | AGACGCACAATCTTGAGAGCAG |  |
